# Maryland State Board of Dental Examiners

**Published:** 1884-05

**Authors:** 


THE BOARD OF DENTAL EXAMINERS.—Drs. E. P. Keech, Chas. E. Duck; Richard Grady and T. S. Waters, of Baltimore, and Edward Nelson, of Frederick, recently appointed by Governor McLane, met for organization Saturday. Dr. Keech was elected President and Dr. Grady Secretary. It is now the duty of all dentists to cause their names and residence or place of business to be registered with the board, and those who were engaged in the practice of dentistry when the bill was passed must verify the statement under oath before a Notary Public or Justice of the Peace. Regular meetings of the board will be held the third Saturdays in June and December, when those who desire to practice dentistry may appear for examination. Any person who wilfully violates the law is subject to fine and imprisonment. The general experience of the workings of laws regulating the practice in various States is that only about one-third of the candidates for examination are granted certificates.

